# Peripheral immune system activity in young psychiatry patients

**DOI:** 10.1016/j.bbih.2025.101143

**Published:** 2025-12-03

**Authors:** Lennart Seizer, Johanna Löchner, Tobias J. Renner

**Affiliations:** a Department of Clinical Psychology and Psychotherapy for Children and Adolescents, Friedrich-Alexander-Universität Erlangen-Nürnberg, Germany; b Department of Child and Adolescent Psychiatry, Psychosomatics and Psychotherapy, University Hospital of Tübingen, Germany; c German Center for Mental Health (DZPG), Tübingen, Germany

**Keywords:** Psychoneuroimmunology, Immunopsychiatry, Children, Adolescents, Immune cells, Inflammation

## Abstract

The interaction between the immune system and the central nervous system has been implicated in the development of psychiatric disorders in adult patients. However, comprehensive data regarding pediatric psychiatry are still lacking. This study aims to describe the distributions of various markers of immune system activity in a large clinical sample of children and adolescents and summarize the immunological profiles associated with different psychiatric disorders. We analyzed blood samples from 1543 patients aged 6–18 years (62 % female), admitted to the Child and Adolescent Psychiatry at the University Hospital of Tübingen between 2014 and 2024. Immune markers such as C-reactive protein and various cell counts and ratios were measured and regressed on psychiatric diagnoses according to the ICD-10 classification. Our findings revealed several distinct immunological profiles linked to specific psychiatric conditions in youth, such as higher CRP levels in patients with severe stress and adjustment disorders. The study underscores the potential role that immune system aberrations may play in mental health disorders and highlights the importance of investigating this link in this age group. However, some inconsistencies with the existing literature were found, such as the lack of association between depression and immune activity, which calls for further research to elucidate these relationships. Future studies should include longitudinal designs to better understand the causal pathways and potential for immune-targeted therapies in pediatric psychiatry.

## 1 Introduction

A growing body of research has highlighted various ways in which the immune system interacts with the central nervous system. Immune mediators from peripheral sources can reach the brain through humoral, neural, and cellular routes and can affect mood regulation, neurocircuitry, and synaptic plasticity (Nusslock et al., 2024; Parsons et al., 2021, Seizer et al., 2023; Seizer and Löchner, 2024; Capuron and Miller, 2011; Dantzer et al., 2008). As a result, dysregulated immune activation and inflammation are believed to play a role in the development of psychiatric disorders. Changes in immune system activity have been intensively studied and reviewed in relation to mental health in adult samples (Khandaker et al., 2021; Yuan et al., 2019). For example, compared to healthy controls, increased levels of systemic inflammation have been found in patients with depressive disorder (Köhler et al., 2017; Haapakoski et al., 2015), bipolar disorder (Munkholm et al., 2013; Modabbernia et al., 2013), post-traumatic stress disorder (Passos et al., 2015), and schizophrenia (Fernandes et al., 2016; Miller et al., 2014). Moreover, pharmacological research indicates that immune system activity may play a causal role in the onset of psychiatric conditions. Various anti-inflammatory medications have shown significant effects in the treatment of psychiatric disorders, including depressive disorder (Kappelmann et al., 2018), bipolar disorder (Rosenblat et al., 2016), and schizophrenia (Çakici et al., 2019). Similar research exists in the field of child and adolescent psychiatry, but studies in this age group remain scarce compared to adult populations (Seizer et al., 2026, Taylor et al., 2024; Colasanto et al., 2020; D'Acunto et al., 2019; Mitchell and Goldstein, 2014).

Some reviews and meta-analyses to date have addressed the relationship between mental health conditions and immune system activity in youth samples (Taylor et al., 2024; Colasanto et al., 2020; Parsons et al., 2021; D'Acunto et al., 2019; Mitchell and Goldstein, 2014; Mills et al., 2013). Therein, potential evidence for aberrant immune functioning was found, such as a positive correlation of proinflammatory cytokines (CRP and IL-6) with depressive symptoms (Colasanto et al., 2020), higher leukocyte counts and increased levels of proinflammatory cytokines (TNF, CRP, and IL-6) in patients with psychotic disorders compared to healthy controls (Taylor et al., 2024), and a proinflammatory state in patients with autism spectrum disorder (Mitchell and Goldstein, 2014). However, these results are not consistent in the literature. For example, D'Acunto et al. (2019) found no difference in the levels of proinflammatory cytokines (TNF-α, IFN-γ, IL-1β, IL-4, IL-6, IL-8, and IL-10) between young patients with depressive disorders and healthy controls; and Parsons et al. (2021) found no difference in the immune cell counts and cytokine levels between patients with anxiety disorders and healthy controls. Furthermore, all meta-analyses noted several limitations, including the small number of studies, limited sample sizes, lack of control for confounding variables, and substantial heterogeneity in the effects for some immune measures (Taylor et al., 2024; Colasanto et al., 2020; Parsons et al., 2021; D'Acunto et al., 2019; Mitchell and Goldstein, 2014; Mills et al., 2013). Currently, large-sample clinical studies are lacking to reinforce the findings (Khandaker et al., 2021). Such studies may provide robust data that can clarify associations between immune system dysfunction and psychiatric disorders, which are often marked by heterogeneity. Moreover, large sample sizes enhance the reliability and generalizability of findings – a key translational goal for research in this field (Leboyer et al., 2016).

There are several reasons why more thorough assessments in the population of children and adolescents are warranted. Firstly, childhood and adolescence are vulnerable phases for the development of mental health problems with about half of all mental health disorders having their onset before the age of 18 and the average age of onset across mental disorders being approximately 14.5 years (Solmi et al., 2022). Thus, a better understanding of the etiology and the development of prevention and treatment strategies for this age group could potentially reduce the severity and duration of psychological illnesses. Additionally, it may help reduce physical illnesses associated with prolonged inflammation and the chronic course of diseases. Secondly, immune system activity, including cytokine production, can vary between children and adults (Valiathan et al., 2016; Vogelzangs et al., 2013) and adolescence is marked by changes in the neuroendocrine and physiological systems, as well as restructuring of the brain networks supporting emotion regulation, socio-emotional processing, and executive function (Cisler and Herringa, 2021). Consequently, research findings from adult studies may not be directly relevant to youth patients. Thirdly, the connection between immune system activity and mental health may be clearer in children and adolescents due to the lower prevalence of medical comorbidities in these younger groups and potentially greater external regulation of lifestyle factors (e.g., physical activity, diet, and hygiene) by parents and teachers (Mitchell and Goldstein, 2014). In the current study, we aim to describe the distributions of various markers of immune system activity in a large clinical sample of children and adolescents and summarize the immunological profiles associated with different psychiatric disorders.

## 2 Methods

### 2.1 Study sample

The sample consists of 1543 children and adolescents (62 % female) who were admitted as patients to the Child and Adolescent Psychiatry at the University Hospital of Tübingen between 2014 and 2024 and who had blood samples taken for immune marker determination. The mean age of patients was 14.52 years (*SD* = 2.51, range = 6.63–18.24) and they received on average two diagnoses (range = 1–6) based on the International Statistical Classification of Diseases and Related Health Problems (ICD-10). The diagnoses were determined by multidisciplinary teams, each including a board-certified child and adolescent psychiatrist and psychotherapist. Individual diagnoses were grouped into higher-order ICD-10 categories. Only diagnoses that occurred in at least 30 patients were included in the analyses. Table 1 presents the frequency of each diagnosis in the sample, along with descriptive statistics. A detailed description of the diagnoses and comorbidity is available in Supplement S1 and S2 (https://osf.io/78cvh). Some patients were admitted multiple times during the observation period, with new blood samples collected at each admission. On average, 1.45 measurements were available per patient (*SD* = 0.90, range = 1–11). Patients with immunological diseases or those receiving immune-modulating medication were excluded from the sample (*n* = 51 patients). The study was approved by the Ethics Committee of the University Hospital of Tübingen.

**Table 1:** Descriptive statistics for the sample, broken down by diagnoses. Note that the sum of diagnoses is higher than the total number of patients, as some patients received multiple diagnoses. BMI = Body mass index. M = Mean. SD = Standard deviation. %F = proportion of females.

ICD	Diagnosis	*N*	Sex (%F)	Age		BMI	
				*M*	*SD*	*M*	*SD*
F12	Mental and behavioral disorders due to use of cannabinoids	34	41	16.53	1.36	20.67	3.58
F19	Mental and behavioral disorders due to multiple drug use	36	59	15.88	1.43	22.49	5.41
F23	Acute and transient psychotic disorders	39	42	16.36	1.27	20.28	2.95
F32	Depressive episode	816	81	15.37	1.66	21.47	5.14
F33	Recurrent depressive disorder	34	85	15.55	1.48	23.23	6.10
F40	Phobic anxiety disorders	183	85	15.54	1.59	22.37	5.64
F41	Other anxiety disorders	85	75	15.12	1.95	21.96	5.75
F42	Obsessive-compulsive disorder	99	61	14.70	2.32	19.52	3.75
F43	Reaction to severe stress, and adjustment disorders	164	77	15.25	2.09	21.83	5.40
F44	Dissociative (conversion) disorders	38	82	15.75	1.39	21.53	4.31
F45	Somatoform disorders	66	71	14.99	1.95	21.77	4.83
F50	Eating disorders	195	96	15.12	1.64	17.87	5.07
F63	Habit and impulse disorders	115	29	14.95	2.04	22.85	7.07
F80	Specific developmental disorders of speech and language	30	41	11.42	1.82	19.98	4.67
F81	Specific developmental disorders of scholastic skills	92	48	13.59	2.72	21.02	5.93
F84	Pervasive developmental disorders	169	33	13.67	2.90	19.82	4.62
F90	Hyperkinetic disorders	263	20	11.78	2.94	19.34	4.72
F91	Conduct disorders	149	21	12.20	2.98	20.63	4.40
F92	Mixed disorders of conduct and emotions	192	50	13.90	2.59	21.77	6.65
F93	Emotional disorders with onset specific to childhood	215	52	12.51	2.53	20.67	5.88
F94	Disorders of social functioning with onset specific to childhood	91	46	13.00	3.12	20.26	4.76
F98	Other behavioral and emotional disorders with onset in childhood	121	34	11.85	2.94	21.20	7.04

### 2.2 Immune markers

Cell counts, including lymphocytes, monocytes, basophils, eosinophils, neutrophils, and total leukocytes, were determined using flow cytometry (Sysmex, XN-9100) in EDTA whole blood samples and are reported as thousands per microliter. CRP concentrations were determined by immunoturbidimetry (BioMajesty, Atellica CH) in blood serum samples. The lower limit of detection for CRP concentrations was 0.01 mg/dl. Additionally, the neutrophil-to-lymphocyte ratio (NLR) was calculated as neutrophils/lymphocytes, the monocyte-to-lymphocyte ratio (MLR) as monocytes/lymphocytes, and the systemic inflammation response index (SIRI) as (neutrophils × monocytes)/lymphocytes (Qi et al., 2016). At the time of blood sampling, probable acute infection status was also assessed through clinical examination. For example, patients presenting with fever or other acute illness symptoms were noted.

### 2.3 Covariates

The same set of covariates was considered in all regression models: (1) age in years, to account for physiological changes in the neuroendocrine and immune systems during development; (2) sex (male or female), since biological differences such as hormonal variation can influence immune responses; and (3) body mass index (BMI), as both obesity and underweight can alter cytokine levels and immune responses. BMI was determined based on weight and height measured by professional clinical personnel during admission, and percentiles were calculated based on age- and sex-matched standards from the Centers for Disease Control and Prevention (CDC) and used in the analysis to account for age- and sex-related growth differences. Sex was assessed by self-report during admission. Four patients did not identify as male or female and were excluded from analyses due to the small group size. All covariates were measured at the time of admission. Distributions of the covariates are presented in Table 1.

### 2.4 Data analysis

All statistical analyses were performed in *R* 4.4 (R Core Team, 2024). Supplemental material is available at https://osf.io/78cvh. T-tests and Wilcoxon-tests were conducted to test sex differences in the immune measures. Multilevel correlations were performed to examine the relationships between age, BMI, and immune measures. These extend standard correlation analyses by accounting for the non-independence of multiple measurements within individuals (Makowski et al., 2019, 2022). To investigate the associations of ICD-10 diagnoses with the different immune markers, multilevel regression models with random intercepts were fitted (Bates and Bolker, 2015). For each immune marker a separate model was fit, with the respective marker as outcome and the diagnoses as categorial predictors (absent or present). CRP values were log-transformed to correct skewness in the residual distribution of models using raw values. The sample size allowed detection of small effects (*f*^*2*^ = 0.02) with over 95 % power at *α* = .05 (Zhang and Mai, 2023). Bonferroni correction was applied to adjust for multiple comparisons and reduce the risk of false positives.

## 3 Results

**Fig. 1:**
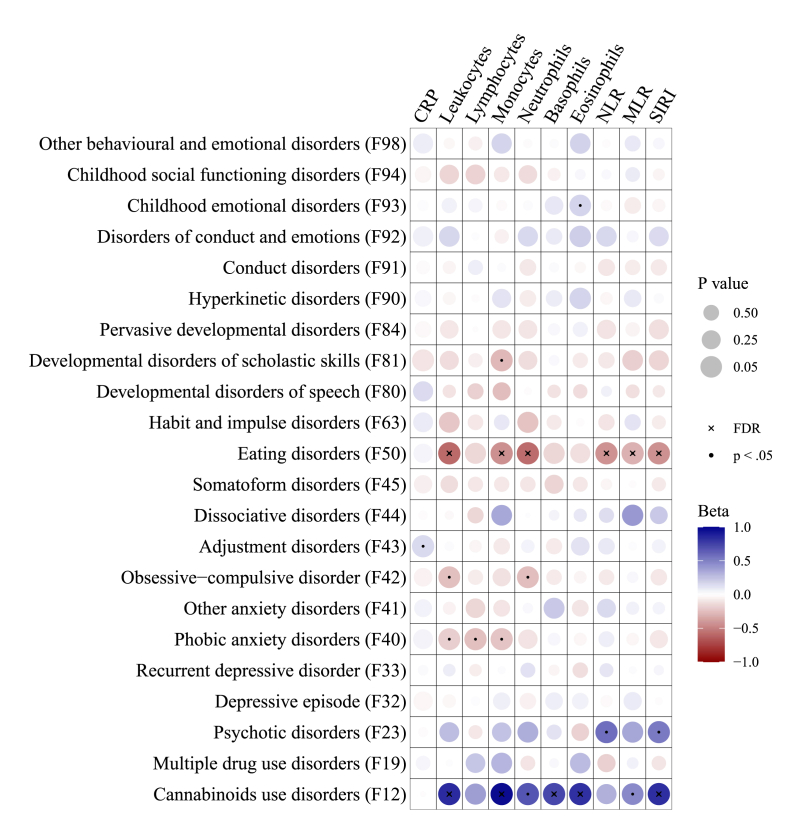
Standardized regression estimates from the multi-level models. Each column shows the model with the respective immune marker as outcome. All models have been adjusted for age, sex, and BMI. The circles' color and size indicate the effect size and significance. Effects that are significant at the p < .05 criterion are marked by a black dot, and effects that remain significant after Bonferroni-correction are marked by a black cross. A detailed description of all test statistics is available in the supplemental material. CRP = C-reactive protein. NLR = Neutrophil-to-lymphocyte ratio. MLR = Monocyte-to-lymphocyte ratio. SIRI = Systemic inflammation response index. (For interpretation of the references to color in this figure legend, the reader is referred to the Web version of this article.)

The associations between immune markers and covariates are summarized in Table 2. Age and BMI were both positively associated with most immune markers. An exception was lymphocyte counts, which decreased with age. Significant sex differences were observed for lymphocytes, monocytes, eosinophils (all higher in males), neutrophils, and NLR (higher in females). No significant associations were found for basophils across any demographic variable. Fig. 1 shows the associations between diagnosis groups and immune markers. In the following, these results are presented in detail, structured by immune markers. We restrict ourselves to the discussion of significant results for the sake of brevity, but a detailed description of all models and test statistics is available in Supplement S3 (https://osf.io/78cvh). Further, the following *p*-values in text represent the uncorrected values, but Fig. 1 highlights which of these effects remained significant after Bonferroni-correction. In sensitivity analyses, we have excluded all data where patients showed signs of acute infection at the time of blood sampling, which led to no change regarding the direction and significance of the results. The sensitivity analyses are available in Supplement S4 (https://osf.io/78cvh).

**Table 2:** Associations between the demographic variables and immune marker. For age and BMI, correlations were calculated. For sex differences, t-tests were computed. The differences in immune measures between males as females are given as the differences of the mean male value minus the mean female value (M-F). CRP = C-reactive protein. NLR = Neutrophil-to-lymphocyte ratio. MLR = Monocyte-to-lymphocyte ratio. SIRI = Systemic inflammation response index.

	Age		BMI		Sex	
	r	p	r	p	M-F	p
CRP	.11	<.001	.11	<.001	.02	.351
Leukocytes	.06	.014	.15	<.001	−.12	.300
Lymphocytes	−.09	<.001	.13	<.001	.13	<.001
Monocytes	.02	.478	.18	<.001	.04	<.001
Neutrophils	.11	<.001	.11	<.001	−.35	<.001
Basophils	−.02	.489	.01	.953	.01	.136
Eosinophils	−.03	.209	.11	<.001	.06	<.001
NLR	.12	<.001	.02	.348	−.23	<.001
MLR	.07	.007	.04	.148	.01	.217
SIRI	.09	<.001	.07	.013	−.05	.280

**C-reactive protein**. After adjustment for age, sex, and BMI, higher CRP concentrations were associated with severe stress and adjustment disorders (F43; *b* = 0.45, 95 % CI [0.14, 0.76], *p* = .004).

**Leukocytes**. Higher leukocyte counts were found in mental and behavioral disorders due to use of cannabinoids (F12; *b* = 1.77, 95 % CI [0.85, 2.68], *p* < .001), while lower leukocyte counts were found in phobic anxiety disorders (F40; *b* = −0.41, 95 % CI [−0.77, −0.04], *p* = .029), obsessive-compulsive disorders (F42; *b* = −0.52, 95 % CI [−0.99, −0.04], *p* = .033), eating disorders (F50; *b* = −1.24, 95 % CI [−1.61, −0.87], *p* < .001).

**Lymphocytes**. Lower lymphocyte counts were associated with phobic anxiety disorders (F40; *b* = −0.17, 95 % CI [−0.28, −0.05], *p* = .005).

**Monocytes**. Higher monocyte counts were associated with mental and behavioral disorders due to use of cannabinoids (F12; *b* = 0.17, 95 % CI [0.09, 0.24], *p* < .001), while lower monocyte counts were associated with phobic anxiety disorders (F40; *b* = −0.04, 95 % CI [−0.07, −0.01], *p* = .009), eating disorders (F50; *b* = −0.08, 95 % CI [−0.11, −0.05], *p* < .001), and developmental disorders of scholastic skills (F81; *b* = −0.05, 95 % CI [−0.09, −0.01], *p* = .017).

**Neutrophils**. Higher neutrophil counts were associated with mental and behavioral disorders due to use of cannabinoids (F12; *b* = 1.19, 95 % CI [0.43, 1.94], *p* = .002), while lower neutrophil counts were associated with obsessive-compulsive disorders (F42; *b* = −0.45, 95 % CI [−0.84, −0.05], *p* = .028), eating disorders (F50; *b* = −1.02, 95 % CI [−1.33, −0.71], *p* < .001).

**Basophils**. Higher basophil counts were associated with mental and behavioral disorders due to use of cannabinoids (F12; *b* = 0.01, 95 % CI [0.01, 0.02], *p* = .001).

**Eosinophils**. Higher eosinophil counts were associated with mental and behavioral disorders due to use of cannabinoids (F12; *b* = 0.14, 95 % CI [0.07, 0.21], *p* < .001) and emotional disorders with onset specific to childhood (F93; *b* = 0.03, 95 % CI [0.00, 0.06], *p* = .048).

**Neutrophil-to-lymphocyte ratio**. A higher NLR was associated with psychotic disorders (F23; *b* = 0.64, 95 % CI [0.20, 1.08], *p* = .005), while a lower NLR was found in eating disorders (F50; *b* = −0.48, 95 % CI [−0.68, −0.28], *p* < .001).

**Monocyte-to-lymphocyte ratio**. A higher MLR was associated with mental and behavioral disorders due to use of cannabinoids (F12; *b* = 0.05, 95 % CI [0.00, 0.09], *p* = .042), while a lower MLR was found in eating disorders (F50; *b* = −0.03, 95 % CI [−0.05, −0.01], *p* = .001).

**Systemic inflammation response index**. A higher SIRI was associated with mental and behavioral disorders due to use of cannabinoids (F12; *b* = 0.72, 95 % CI [0.33, 1.10], *p* < .001) and psychotic disorders (F23; *b* = 0.45, 95 % CI [0.11, 0.80], *p* = .010), while lower SIRI was found in eating disorders (F50; *b* = −0.38, 95 % CI [−0.53, −0.22], *p* < .001).

## 4 Discussion

In this study, we compared the distributions of peripheral inflammation and immune cell counts across various psychiatric conditions in a large clinical sample of children and adolescents. We found several significant differences in immune system variables associated with specific diagnoses, which may provide promising avenues for future research and clinical applications. Importantly, our results often diverge from previous findings in adult populations, highlighting that we cannot directly extrapolate findings from adults to youth. Children and adolescents undergo distinct biological developmental processes, and it is important to investigate the distinct characteristics of this population, especially as most mental health disorders have their onset during childhood and adolescence (Solmi et al., 2022).

Higher levels of systemic inflammation (CRP) were found in patients with severe stress and adjustment disorders. This result is consistent with studies in adult populations where PTSD has been characterized by increased pro-inflammatory and decreased anti-inflammatory cytokine levels (Yang and Jiang, 2020; Yuan et al., 2019; Passos et al., 2015; Eraly et al., 2014) and to heightened IL-6 production following stimulation (Rohleder et al., 2004). Further, a previous meta-analysis on anxiety disorders reported increased inflammation only in individuals with PTSD, but not in other anxiety-related disorders (Renna et al., 2018). However, it is currently unclear whether pre-existing or trauma-induced inflammation increases the risk of developing PTSD, or whether the inflammatory milieu is a result of PTSD (Katrinli et al., 2022).

Phobic anxiety disorders (F40) and obsessive-compulsive disorders (F42) were both associated with lower leukocyte counts. Specifically, phobic anxiety disorders showed decreased numbers of lymphocytes and monocytes, and obsessive-compulsive disorders were associated with decreased numbers of neutrophils. Previous studies on these associations have produced mixed results. A study on adult patients with panic disorder and agoraphobia found decreased levels of circulating CD19^+^ B lymphocytes compared to healthy controls (Schleifer et al., 2002), while other studies found increased numbers of leukocytes in child and adult patients with anxiety-related disorders (Uzun and Akıncı, 2021; Özyurt and Binici, 2019). A recent meta-analysis on anxiety-related disorders found a non-significant decrease of lymphocytes in patients compared to healthy controls (Parsons et al., 2021). Regarding obsessive-compulsive disorders, our results are consistent with the literature with previous studies finding decreased levels of leukocytes and neutrophils in child and adult patients compared to healthy controls (Uzun et al., 2020; Özyurt and Binici, 2019; Atmaca et al., 2011).

Patients with eating disorders (F50) showed lower levels across several immune cell counts and ratios. This comes as no surprise, as these patients exhibit abnormal eating habits, which may lead to malnutrition. The functionality of the immune system depends on the availability of nutrients and deficiencies can impair immune function (Keeler et al., 2023; Straub et al., 2010; Marcos, 2000). Mental and behavioral disorders due to use of cannabinoids (F12) were associated with higher immune cell counts and ratios. A previous study found a similar increase in leukocytes in heavy cannabis users compared to never users, but not in occasional cannabis users (Alshaarawy et al., 2019). Additionally, there is a well-established link between increased leukocyte counts and tobacco use, which is often connected to cannabis use (Higuchi et al., 2016). Our data do not allow us to determine whether the increase in immune cell counts is a pharmacological consequence of cannabis and tobacco use or a psychophysiological feature of the addiction disorder.

Psychotic disorders (F23) were associated with elevated NLR and SIRI. These findings are in line with previous meta-analyses that have consistently reported proinflammatory states in pediatric psychotic disorders (Taylor et al., 2024). Our findings confirm and extend these associations in pediatric populations and suggest that inflammatory processes may be active early in the course of psychotic illness. Moreover, elevated NLR and SIRI in psychotic youth align with recent research indicating a heightened peripheral immune response, possibly reflecting a proinflammatory state that contributes to or results from the underlying pathophysiology of psychosis (Chen et al., 2024).

To our surprise, we found no association between immune system activity and depressive disorders, which is arguably the most prevalent link in immunopsychiatry established in adult populations (Yuan et al., 2019). There has been research questioning whether child-, adolescent-, and adult-onset depression share the same biological correlates (Kaufman et al., 2001). Vogelzangs et al. (2013) found the increase in CRP in patients with anxiety disorders compared to healthy controls to be moderated by the patients' age of onset, with higher ages being related to a more pronounced increase in CRP. Further, our results are consistent with a previous meta-analysis that found no significant difference in cytokine levels (TNF-α, IFN-γ, IL-1β, IL-4, IL-6, IL-8, and IL-10) between children and adolescents with depressive disorder versus healthy controls (D'Acunto et al., 2019). However, another meta-analysis that investigated the correlation between the severity of depression symptoms and cytokines in both, clinical and community samples, found a positive association with CRP and IL-6 (Colasanto et al., 2020). This points to the existing hypothesis that immune system activity may be related to depression in a symptom-specific manner: Previous studies found higher levels of inflammatory markers to be mainly associated with neurovegetative symptoms like fatigue, appetite, and sleep problems (Milaneschi et al., 2021; Jokela et al., 2016). Thus, the relation between depression and inflammation may be more pronounced when examined in relation to symptom severity scores rather than a diagnosis-based approach, given the many heterogeneous clinical presentations of depression (Goldberg, 2011; Fried, 2017).

The present study has several limitations. First, additional cytokines and sub-populations of the immune cell groups could have been measured to provide further insights into immune system activity. For example, different pro-inflammatory cytokines and lymphocyte subtypes have shown differential associations with mental health conditions in previous studies (Yuan et al., 2019). The current analyses are based on different diagnostic groups within a clinical population without the inclusion of a healthy control group. Thus, systematic differences in outcome variables across all patients could limit the comparability to healthy individuals. Further, exclusively focusing on a clinical population limits generalizability as only a part of youth with mental health disorders (about one-third) are in contact with mental health care providers (Georgiades et al., 2019). Another limitation of our study is that information on the duration since symptom onset was not available. Diagnoses were recorded during admission and therefore reflect the patients’ current clinical status at the time of blood sampling. However, we cannot rule out that illness duration may have affected immune system activity, and future studies should take this into account. Moreover, symptom-level analyses using data from condition-specific instruments may provide further insights into potential specificities of the link with immune system activity (Schubert et al., 2021). For example, in depression, inflammation has mainly been associated with neurovegetative symptoms such as fatigue, appetite and sleep problems (e.g., Jokela et al., 2016; Milaneschi et al., 2021; Seizer et al., 2024). Another limitation is that pubertal status, which can influence immune function and mental health via hormonal and neurodevelopmental changes, was not assessed. Although we adjusted for age, pubertal stage may represent an important covariate for future studies.

## 5 Conclusion

This study reveals significant associations between immune system activity and various psychiatric disorders in children and adolescents. The findings underscore the distinct immunological profiles linked to specific diagnoses and highlight the importance of investigating immune markers in this age group separately from adults. These results point to opportunities for research on immune-targeted treatments tailored to the specific psychiatric conditions in youth (Nusslock et al., 2024). However, the study also found some inconsistencies with existing literature, for example, regarding depression and immune system activity, suggesting the need for further exploration. Future longitudinal studies are needed to better understand the causal relationships and long-term implications of immune system dysregulation in psychiatric disorders among children and adolescents (Seizer and Schubert, 2024; Löchner et al., 2024).

## CRediT authorship contribution statement

**Lennart Seizer:** Writing – original draft, Formal analysis. **Johanna Löchner:** Writing – review & editing. **Tobias J. Renner:** Writing – review & editing.

## Financial disclosures

The authors declare no conflicts of interest.

## Declaration of competing interest

The authors declare no conflicts of interest.

## Data Availability

Data will be made available on request.
