# Supplementary material for: Peripheral immune system activity in young psychiatry patients
Source: Brain Behav Immun Health. 2025 Dec 3;52:101143. doi: 10.1016/j.bbih.2025.101143 (PMC12816897; doi:10.1016/j.bbih.2025.101143)
Supplement: Multimedia component 1 [file mmc1.zip › mmc1.html]

Supplement


# Supplement

### C-reactive protein

| effect | term | estimate | std.error | statistic | df | p.value | conf.low | conf.high |
| --- | --- | --- | --- | --- | --- | --- | --- | --- |
| fixed | (Intercept) | -6.561 | 0.336 | -19.529 | 1099.417 | 0.000 | -7.221 | -5.902 |
| fixed | F121 | -0.080 | 0.304 | -0.264 | 1142.166 | 0.792 | -0.678 | 0.517 |
| fixed | F191 | 0.009 | 0.257 | 0.033 | 1263.484 | 0.974 | -0.495 | 0.512 |
| fixed | F231 | -0.043 | 0.298 | -0.145 | 1257.380 | 0.885 | -0.627 | 0.541 |
| fixed | F321 | -0.116 | 0.105 | -1.105 | 1260.389 | 0.269 | -0.322 | 0.090 |
| fixed | F331 | -0.150 | 0.309 | -0.486 | 1264.402 | 0.627 | -0.756 | 0.456 |
| fixed | F401 | 0.196 | 0.138 | 1.423 | 1250.851 | 0.155 | -0.074 | 0.467 |
| fixed | F411 | 0.131 | 0.202 | 0.645 | 1200.047 | 0.519 | -0.267 | 0.528 |
| fixed | F421 | -0.146 | 0.173 | -0.843 | 1234.230 | 0.399 | -0.486 | 0.194 |
| fixed | F431 | 0.439 | 0.155 | 2.823 | 1264.811 | 0.005 | 0.134 | 0.743 |
| fixed | F441 | 0.133 | 0.276 | 0.480 | 1219.129 | 0.631 | -0.409 | 0.674 |
| fixed | F451 | -0.228 | 0.218 | -1.047 | 1243.429 | 0.295 | -0.655 | 0.199 |
| fixed | F501 | 0.142 | 0.138 | 1.026 | 1132.808 | 0.305 | -0.130 | 0.414 |
| fixed | F601 | 0.175 | 0.237 | 0.738 | 1226.854 | 0.461 | -0.290 | 0.639 |
| fixed | F631 | 0.287 | 0.214 | 1.344 | 1223.064 | 0.179 | -0.132 | 0.706 |
| fixed | F801 | 0.955 | 0.373 | 2.563 | 1088.054 | 0.011 | 0.224 | 1.686 |
| fixed | F811 | -0.298 | 0.176 | -1.693 | 1225.343 | 0.091 | -0.643 | 0.047 |
| fixed | F841 | -0.107 | 0.128 | -0.843 | 1218.657 | 0.400 | -0.358 | 0.143 |
| fixed | F901 | 0.135 | 0.133 | 1.017 | 1200.839 | 0.309 | -0.125 | 0.395 |
| fixed | F911 | -0.001 | 0.161 | -0.008 | 1245.927 | 0.994 | -0.318 | 0.315 |
| fixed | F921 | 0.252 | 0.150 | 1.683 | 1235.613 | 0.093 | -0.042 | 0.546 |
| fixed | F931 | -0.044 | 0.131 | -0.338 | 1262.496 | 0.736 | -0.301 | 0.212 |
| fixed | F941 | -0.040 | 0.195 | -0.206 | 1166.881 | 0.837 | -0.423 | 0.343 |
| fixed | F981 | 0.117 | 0.180 | 0.649 | 1241.282 | 0.517 | -0.236 | 0.470 |
| fixed | age | 0.142 | 0.021 | 6.792 | 1095.484 | 0.000 | 0.101 | 0.183 |
| fixed | bmi | 0.017 | 0.001 | 12.236 | 1072.488 | 0.000 | 0.014 | 0.019 |
| fixed | sexMALE | 0.206 | 0.109 | 1.897 | 975.906 | 0.058 | -0.007 | 0.420 |

### Leucocytes

| effect | term | estimate | std.error | statistic | df | p.value | conf.low | conf.high |
| --- | --- | --- | --- | --- | --- | --- | --- | --- |
| fixed | (Intercept) | 6.187 | 0.469 | 13.184 | 1253.184 | 0.000 | 5.266 | 7.108 |
| fixed | F121 | 1.423 | 0.450 | 3.159 | 1299.405 | 0.002 | 0.539 | 2.306 |
| fixed | F191 | 0.016 | 0.368 | 0.044 | 1462.028 | 0.965 | -0.706 | 0.738 |
| fixed | F231 | 0.564 | 0.409 | 1.381 | 1423.117 | 0.167 | -0.237 | 1.366 |
| fixed | F321 | -0.055 | 0.144 | -0.383 | 1450.763 | 0.702 | -0.338 | 0.228 |
| fixed | F331 | 0.236 | 0.403 | 0.585 | 1452.078 | 0.559 | -0.554 | 1.026 |
| fixed | F401 | -0.418 | 0.183 | -2.277 | 1459.215 | 0.023 | -0.777 | -0.058 |
| fixed | F411 | -0.122 | 0.262 | -0.466 | 1333.363 | 0.641 | -0.635 | 0.391 |
| fixed | F421 | -0.518 | 0.235 | -2.202 | 1395.267 | 0.028 | -0.980 | -0.057 |
| fixed | F431 | 0.058 | 0.212 | 0.275 | 1461.378 | 0.784 | -0.358 | 0.474 |
| fixed | F441 | -0.299 | 0.408 | -0.733 | 1402.256 | 0.464 | -1.100 | 0.502 |
| fixed | F451 | -0.357 | 0.298 | -1.198 | 1423.977 | 0.231 | -0.941 | 0.227 |
| fixed | F501 | -1.257 | 0.188 | -6.672 | 1275.043 | 0.000 | -1.627 | -0.887 |
| fixed | F601 | -0.783 | 0.346 | -2.266 | 1417.310 | 0.024 | -1.462 | -0.105 |
| fixed | F631 | -0.393 | 0.281 | -1.399 | 1445.366 | 0.162 | -0.945 | 0.158 |
| fixed | F801 | 0.099 | 0.519 | 0.191 | 1231.472 | 0.849 | -0.920 | 1.118 |
| fixed | F811 | -0.286 | 0.242 | -1.182 | 1407.611 | 0.237 | -0.760 | 0.188 |
| fixed | F841 | -0.264 | 0.177 | -1.490 | 1419.638 | 0.137 | -0.611 | 0.084 |
| fixed | F901 | 0.013 | 0.184 | 0.070 | 1349.299 | 0.944 | -0.348 | 0.374 |
| fixed | F911 | -0.002 | 0.224 | -0.011 | 1442.397 | 0.992 | -0.441 | 0.437 |
| fixed | F921 | 0.285 | 0.212 | 1.343 | 1434.454 | 0.179 | -0.131 | 0.700 |
| fixed | F931 | 0.119 | 0.179 | 0.665 | 1467.006 | 0.506 | -0.233 | 0.471 |
| fixed | F941 | -0.296 | 0.272 | -1.089 | 1360.956 | 0.276 | -0.829 | 0.237 |
| fixed | F981 | -0.046 | 0.242 | -0.192 | 1445.967 | 0.848 | -0.520 | 0.427 |
| fixed | age | 0.050 | 0.029 | 1.715 | 1240.172 | 0.087 | -0.007 | 0.107 |
| fixed | bmi | 0.010 | 0.002 | 5.235 | 1199.936 | 0.000 | 0.006 | 0.014 |
| fixed | sexMALE | -0.384 | 0.151 | -2.534 | 1070.535 | 0.011 | -0.681 | -0.087 |

### Lymphocytes

| effect | term | estimate | std.error | statistic | df | p.value | conf.low | conf.high |
| --- | --- | --- | --- | --- | --- | --- | --- | --- |
| fixed | (Intercept) | 3.065 | 0.151 | 20.340 | 1290.481 | 0.000 | 2.769 | 3.360 |
| fixed | F121 | 0.216 | 0.143 | 1.510 | 1362.431 | 0.131 | -0.065 | 0.497 |
| fixed | F191 | 0.156 | 0.115 | 1.352 | 1459.833 | 0.176 | -0.070 | 0.382 |
| fixed | F231 | -0.057 | 0.126 | -0.453 | 1334.590 | 0.651 | -0.305 | 0.190 |
| fixed | F321 | -0.001 | 0.045 | -0.028 | 1462.977 | 0.978 | -0.090 | 0.088 |
| fixed | F331 | -0.036 | 0.125 | -0.290 | 1397.173 | 0.772 | -0.281 | 0.209 |
| fixed | F401 | -0.166 | 0.057 | -2.882 | 1461.370 | 0.004 | -0.279 | -0.053 |
| fixed | F411 | -0.111 | 0.083 | -1.341 | 1381.072 | 0.180 | -0.274 | 0.052 |
| fixed | F421 | -0.059 | 0.074 | -0.799 | 1446.642 | 0.424 | -0.205 | 0.086 |
| fixed | F431 | -0.023 | 0.067 | -0.342 | 1462.902 | 0.732 | -0.154 | 0.108 |
| fixed | F441 | -0.147 | 0.129 | -1.140 | 1446.267 | 0.254 | -0.400 | 0.106 |
| fixed | F451 | -0.084 | 0.094 | -0.896 | 1448.852 | 0.370 | -0.268 | 0.100 |
| fixed | F501 | -0.112 | 0.060 | -1.868 | 1359.772 | 0.062 | -0.230 | 0.006 |
| fixed | F601 | -0.090 | 0.107 | -0.841 | 1263.371 | 0.400 | -0.299 | 0.119 |
| fixed | F631 | -0.035 | 0.088 | -0.398 | 1462.738 | 0.691 | -0.208 | 0.138 |
| fixed | F801 | 0.011 | 0.166 | 0.067 | 1226.956 | 0.947 | -0.315 | 0.337 |
| fixed | F811 | -0.044 | 0.076 | -0.575 | 1432.764 | 0.565 | -0.194 | 0.106 |
| fixed | F841 | -0.030 | 0.056 | -0.538 | 1455.580 | 0.590 | -0.140 | 0.080 |
| fixed | F901 | 0.008 | 0.059 | 0.134 | 1399.167 | 0.894 | -0.107 | 0.123 |
| fixed | F911 | 0.056 | 0.071 | 0.799 | 1455.889 | 0.424 | -0.082 | 0.195 |
| fixed | F921 | -0.014 | 0.067 | -0.215 | 1460.672 | 0.830 | -0.146 | 0.117 |
| fixed | F931 | 0.020 | 0.056 | 0.360 | 1460.014 | 0.719 | -0.090 | 0.131 |
| fixed | F941 | -0.131 | 0.086 | -1.519 | 1401.843 | 0.129 | -0.300 | 0.038 |
| fixed | F981 | -0.016 | 0.076 | -0.206 | 1456.640 | 0.837 | -0.165 | 0.134 |
| fixed | age | -0.061 | 0.009 | -6.557 | 1274.009 | 0.000 | -0.080 | -0.043 |
| fixed | bmi | 0.002 | 0.001 | 4.001 | 1250.589 | 0.000 | 0.001 | 0.004 |
| fixed | sexMALE | -0.034 | 0.049 | -0.695 | 1092.781 | 0.487 | -0.130 | 0.062 |

### Monocytes

| effect | term | estimate | std.error | statistic | df | p.value | conf.low | conf.high |
| --- | --- | --- | --- | --- | --- | --- | --- | --- |
| fixed | (Intercept) | 0.455 | 0.039 | 11.585 | 1288.835 | 0.000 | 0.378 | 0.532 |
| fixed | F121 | 0.144 | 0.037 | 3.846 | 1362.496 | 0.000 | 0.070 | 0.217 |
| fixed | F191 | 0.045 | 0.030 | 1.495 | 1460.538 | 0.135 | -0.014 | 0.104 |
| fixed | F231 | 0.045 | 0.033 | 1.353 | 1331.904 | 0.176 | -0.020 | 0.109 |
| fixed | F321 | 0.011 | 0.012 | 0.936 | 1463.990 | 0.349 | -0.012 | 0.034 |
| fixed | F331 | 0.013 | 0.033 | 0.391 | 1395.986 | 0.696 | -0.051 | 0.077 |
| fixed | F401 | -0.038 | 0.015 | -2.504 | 1462.178 | 0.012 | -0.067 | -0.008 |
| fixed | F411 | -0.016 | 0.022 | -0.737 | 1381.306 | 0.461 | -0.059 | 0.027 |
| fixed | F421 | -0.025 | 0.019 | -1.302 | 1447.862 | 0.193 | -0.063 | 0.013 |
| fixed | F431 | -0.012 | 0.017 | -0.716 | 1463.862 | 0.474 | -0.047 | 0.022 |
| fixed | F441 | 0.025 | 0.034 | 0.754 | 1447.429 | 0.451 | -0.041 | 0.091 |
| fixed | F451 | -0.019 | 0.024 | -0.782 | 1449.938 | 0.434 | -0.067 | 0.029 |
| fixed | F501 | -0.076 | 0.016 | -4.831 | 1359.874 | 0.000 | -0.106 | -0.045 |
| fixed | F601 | -0.039 | 0.028 | -1.417 | 1258.770 | 0.157 | -0.094 | 0.015 |
| fixed | F631 | 0.021 | 0.023 | 0.912 | 1463.793 | 0.362 | -0.024 | 0.066 |
| fixed | F801 | -0.021 | 0.043 | -0.491 | 1224.341 | 0.624 | -0.106 | 0.064 |
| fixed | F811 | -0.047 | 0.020 | -2.341 | 1433.632 | 0.019 | -0.086 | -0.008 |
| fixed | F841 | -0.021 | 0.015 | -1.437 | 1456.788 | 0.151 | -0.050 | 0.008 |
| fixed | F901 | 0.022 | 0.015 | 1.436 | 1399.662 | 0.151 | -0.008 | 0.052 |
| fixed | F911 | 0.011 | 0.018 | 0.578 | 1456.964 | 0.563 | -0.025 | 0.047 |
| fixed | F921 | -0.008 | 0.017 | -0.456 | 1461.797 | 0.649 | -0.042 | 0.026 |
| fixed | F931 | -0.005 | 0.015 | -0.314 | 1460.768 | 0.754 | -0.033 | 0.024 |
| fixed | F941 | -0.008 | 0.022 | -0.350 | 1402.313 | 0.726 | -0.052 | 0.036 |
| fixed | F981 | 0.032 | 0.020 | 1.618 | 1457.709 | 0.106 | -0.007 | 0.071 |
| fixed | age | 0.002 | 0.002 | 0.883 | 1272.399 | 0.378 | -0.003 | 0.007 |
| fixed | bmi | 0.001 | 0.000 | 4.624 | 1249.168 | 0.000 | 0.000 | 0.001 |
| fixed | sexMALE | 0.011 | 0.013 | 0.879 | 1088.729 | 0.380 | -0.014 | 0.036 |

### Neutrophiles

| effect | term | estimate | std.error | statistic | df | p.value | conf.low | conf.high |
| --- | --- | --- | --- | --- | --- | --- | --- | --- |
| fixed | (Intercept) | 2.484 | 0.391 | 6.356 | 1226.638 | 0.000 | 1.717 | 3.251 |
| fixed | F121 | 0.919 | 0.374 | 2.460 | 1258.659 | 0.014 | 0.186 | 1.653 |
| fixed | F191 | -0.181 | 0.308 | -0.589 | 1440.708 | 0.556 | -0.785 | 0.422 |
| fixed | F231 | 0.574 | 0.344 | 1.670 | 1447.092 | 0.095 | -0.100 | 1.249 |
| fixed | F321 | -0.072 | 0.121 | -0.601 | 1426.645 | 0.548 | -0.309 | 0.164 |
| fixed | F331 | 0.270 | 0.338 | 0.797 | 1459.502 | 0.425 | -0.394 | 0.933 |
| fixed | F401 | -0.206 | 0.153 | -1.341 | 1433.455 | 0.180 | -0.506 | 0.095 |
| fixed | F411 | 0.028 | 0.217 | 0.129 | 1299.501 | 0.898 | -0.399 | 0.454 |
| fixed | F421 | -0.427 | 0.196 | -2.180 | 1348.835 | 0.029 | -0.811 | -0.043 |
| fixed | F431 | 0.109 | 0.178 | 0.612 | 1437.344 | 0.541 | -0.240 | 0.458 |
| fixed | F441 | -0.163 | 0.340 | -0.478 | 1363.974 | 0.632 | -0.830 | 0.504 |
| fixed | F451 | -0.232 | 0.248 | -0.934 | 1401.082 | 0.351 | -0.719 | 0.255 |
| fixed | F501 | -1.030 | 0.156 | -6.594 | 1222.458 | 0.000 | -1.337 | -0.724 |
| fixed | F601 | -0.665 | 0.291 | -2.288 | 1456.888 | 0.022 | -1.235 | -0.095 |
| fixed | F631 | -0.374 | 0.235 | -1.595 | 1415.674 | 0.111 | -0.835 | 0.086 |
| fixed | F801 | 0.125 | 0.430 | 0.292 | 1234.273 | 0.771 | -0.719 | 0.970 |
| fixed | F811 | -0.230 | 0.201 | -1.144 | 1387.965 | 0.253 | -0.625 | 0.165 |
| fixed | F841 | -0.209 | 0.148 | -1.412 | 1382.707 | 0.158 | -0.500 | 0.081 |
| fixed | F901 | -0.054 | 0.154 | -0.348 | 1309.970 | 0.728 | -0.355 | 0.248 |
| fixed | F911 | -0.085 | 0.187 | -0.454 | 1428.046 | 0.650 | -0.453 | 0.283 |
| fixed | F921 | 0.231 | 0.178 | 1.298 | 1408.757 | 0.195 | -0.118 | 0.580 |
| fixed | F931 | 0.049 | 0.151 | 0.324 | 1452.816 | 0.746 | -0.247 | 0.345 |
| fixed | F941 | -0.191 | 0.226 | -0.847 | 1329.811 | 0.397 | -0.635 | 0.252 |
| fixed | F981 | -0.035 | 0.203 | -0.174 | 1435.138 | 0.862 | -0.433 | 0.362 |
| fixed | age | 0.108 | 0.024 | 4.463 | 1214.327 | 0.000 | 0.061 | 0.156 |
| fixed | bmi | 0.007 | 0.002 | 4.204 | 1168.296 | 0.000 | 0.004 | 0.010 |
| fixed | sexMALE | -0.405 | 0.125 | -3.239 | 1054.860 | 0.001 | -0.650 | -0.160 |

### Basophiles

| effect | term | estimate | std.error | statistic | df | p.value | conf.low | conf.high |
| --- | --- | --- | --- | --- | --- | --- | --- | --- |
| fixed | (Intercept) | 0.048 | 0.004 | 10.890 | 1356.997 | 0.000 | 0.040 | 0.057 |
| fixed | F121 | 0.013 | 0.004 | 3.197 | 1435.711 | 0.001 | 0.005 | 0.022 |
| fixed | F191 | 0.000 | 0.003 | 0.147 | 1404.533 | 0.883 | -0.006 | 0.007 |
| fixed | F231 | 0.002 | 0.004 | 0.696 | 1213.473 | 0.487 | -0.004 | 0.009 |
| fixed | F321 | 0.001 | 0.001 | 0.876 | 1434.787 | 0.381 | -0.001 | 0.004 |
| fixed | F331 | 0.001 | 0.004 | 0.151 | 1295.767 | 0.880 | -0.006 | 0.007 |
| fixed | F401 | 0.001 | 0.002 | 0.388 | 1407.340 | 0.698 | -0.003 | 0.004 |
| fixed | F411 | 0.004 | 0.002 | 1.712 | 1440.321 | 0.087 | -0.001 | 0.009 |
| fixed | F421 | -0.002 | 0.002 | -0.737 | 1454.957 | 0.461 | -0.006 | 0.003 |
| fixed | F431 | -0.001 | 0.002 | -0.678 | 1429.673 | 0.498 | -0.005 | 0.002 |
| fixed | F441 | -0.001 | 0.004 | -0.275 | 1457.092 | 0.783 | -0.008 | 0.006 |
| fixed | F451 | -0.004 | 0.003 | -1.442 | 1459.780 | 0.150 | -0.009 | 0.001 |
| fixed | F501 | -0.003 | 0.002 | -1.901 | 1442.905 | 0.057 | -0.007 | 0.000 |
| fixed | F601 | -0.002 | 0.003 | -0.810 | 1075.766 | 0.418 | -0.008 | 0.003 |
| fixed | F631 | -0.001 | 0.003 | -0.422 | 1431.409 | 0.673 | -0.006 | 0.004 |
| fixed | F801 | 0.000 | 0.005 | -0.036 | 1266.322 | 0.971 | -0.010 | 0.009 |
| fixed | F811 | 0.001 | 0.002 | 0.432 | 1459.565 | 0.666 | -0.003 | 0.005 |
| fixed | F841 | 0.000 | 0.002 | 0.162 | 1449.681 | 0.871 | -0.003 | 0.003 |
| fixed | F901 | 0.002 | 0.002 | 1.069 | 1452.684 | 0.285 | -0.002 | 0.005 |
| fixed | F911 | 0.001 | 0.002 | 0.309 | 1458.587 | 0.757 | -0.003 | 0.005 |
| fixed | F921 | 0.001 | 0.002 | 0.776 | 1444.011 | 0.438 | -0.002 | 0.005 |
| fixed | F931 | 0.002 | 0.002 | 1.026 | 1415.332 | 0.305 | -0.002 | 0.005 |
| fixed | F941 | -0.001 | 0.002 | -0.551 | 1448.691 | 0.581 | -0.006 | 0.004 |
| fixed | F981 | 0.001 | 0.002 | 0.263 | 1458.790 | 0.792 | -0.004 | 0.005 |
| fixed | age | 0.000 | 0.000 | -1.750 | 1341.421 | 0.080 | -0.001 | 0.000 |
| fixed | bmi | 0.000 | 0.000 | 0.774 | 1335.592 | 0.439 | 0.000 | 0.000 |
| fixed | sexMALE | -0.001 | 0.001 | -0.505 | 1145.682 | 0.614 | -0.004 | 0.002 |

### Eosinophiles

| effect | term | estimate | std.error | statistic | df | p.value | conf.low | conf.high |
| --- | --- | --- | --- | --- | --- | --- | --- | --- |
| fixed | (Intercept) | 0.219 | 0.038 | 5.746 | 1288.683 | 0.000 | 0.144 | 0.294 |
| fixed | F121 | 0.126 | 0.036 | 3.485 | 1369.675 | 0.001 | 0.055 | 0.197 |
| fixed | F191 | 0.040 | 0.029 | 1.398 | 1432.560 | 0.162 | -0.016 | 0.097 |
| fixed | F231 | -0.031 | 0.032 | -0.963 | 1304.957 | 0.336 | -0.095 | 0.032 |
| fixed | F321 | 0.010 | 0.011 | 0.854 | 1443.622 | 0.393 | -0.013 | 0.032 |
| fixed | F331 | -0.015 | 0.032 | -0.472 | 1366.755 | 0.637 | -0.077 | 0.047 |
| fixed | F401 | -0.008 | 0.014 | -0.527 | 1439.344 | 0.598 | -0.036 | 0.021 |
| fixed | F411 | -0.015 | 0.021 | -0.721 | 1386.630 | 0.471 | -0.056 | 0.026 |
| fixed | F421 | -0.007 | 0.019 | -0.368 | 1447.041 | 0.713 | -0.044 | 0.030 |
| fixed | F431 | 0.017 | 0.017 | 0.991 | 1446.515 | 0.322 | -0.017 | 0.050 |
| fixed | F441 | 0.013 | 0.033 | 0.376 | 1418.483 | 0.707 | -0.053 | 0.078 |
| fixed | F451 | 0.002 | 0.025 | 0.091 | 1420.701 | 0.928 | -0.046 | 0.050 |
| fixed | F501 | -0.022 | 0.015 | -1.467 | 1363.227 | 0.143 | -0.052 | 0.008 |
| fixed | F601 | 0.031 | 0.027 | 1.153 | 1180.552 | 0.249 | -0.022 | 0.084 |
| fixed | F631 | -0.003 | 0.022 | -0.149 | 1445.391 | 0.881 | -0.047 | 0.040 |
| fixed | F801 | -0.019 | 0.042 | -0.446 | 1209.518 | 0.656 | -0.101 | 0.064 |
| fixed | F811 | -0.014 | 0.019 | -0.738 | 1429.787 | 0.461 | -0.052 | 0.023 |
| fixed | F841 | 0.008 | 0.014 | 0.599 | 1447.049 | 0.549 | -0.019 | 0.036 |
| fixed | F901 | 0.031 | 0.015 | 2.101 | 1402.240 | 0.036 | 0.002 | 0.060 |
| fixed | F911 | -0.004 | 0.018 | -0.232 | 1446.253 | 0.817 | -0.039 | 0.031 |
| fixed | F921 | 0.034 | 0.017 | 2.013 | 1447.966 | 0.044 | 0.001 | 0.067 |
| fixed | F931 | 0.028 | 0.014 | 1.959 | 1435.886 | 0.050 | 0.000 | 0.055 |
| fixed | F941 | 0.004 | 0.022 | 0.175 | 1403.671 | 0.861 | -0.039 | 0.046 |
| fixed | F981 | 0.034 | 0.019 | 1.798 | 1446.306 | 0.072 | -0.003 | 0.072 |
| fixed | age | -0.007 | 0.002 | -3.010 | 1271.755 | 0.003 | -0.012 | -0.002 |
| fixed | bmi | 0.001 | 0.000 | 3.557 | 1251.581 | 0.000 | 0.000 | 0.001 |
| fixed | sexMALE | 0.038 | 0.012 | 3.032 | 1074.716 | 0.002 | 0.013 | 0.062 |

### NLR

| effect | term | estimate | std.error | statistic | df | p.value | conf.low | conf.high |
| --- | --- | --- | --- | --- | --- | --- | --- | --- |
| fixed | (Intercept) | 0.663 | 0.251 | 2.636 | 1207.965 | 0.009 | 0.169 | 1.156 |
| fixed | F121 | 0.246 | 0.241 | 1.024 | 1215.207 | 0.306 | -0.226 | 0.718 |
| fixed | F191 | -0.219 | 0.200 | -1.093 | 1398.812 | 0.275 | -0.612 | 0.174 |
| fixed | F231 | 0.639 | 0.226 | 2.832 | 1462.552 | 0.005 | 0.197 | 1.082 |
| fixed | F321 | -0.005 | 0.078 | -0.058 | 1386.170 | 0.953 | -0.158 | 0.149 |
| fixed | F331 | 0.133 | 0.222 | 0.598 | 1462.036 | 0.550 | -0.303 | 0.568 |
| fixed | F401 | 0.057 | 0.100 | 0.571 | 1388.609 | 0.568 | -0.139 | 0.253 |
| fixed | F411 | 0.154 | 0.140 | 1.102 | 1263.364 | 0.271 | -0.121 | 0.430 |
| fixed | F421 | -0.073 | 0.127 | -0.575 | 1278.260 | 0.565 | -0.321 | 0.176 |
| fixed | F431 | 0.103 | 0.116 | 0.886 | 1405.307 | 0.376 | -0.125 | 0.330 |
| fixed | F441 | 0.096 | 0.220 | 0.435 | 1312.896 | 0.664 | -0.336 | 0.527 |
| fixed | F451 | -0.038 | 0.161 | -0.233 | 1376.844 | 0.816 | -0.354 | 0.279 |
| fixed | F501 | -0.481 | 0.101 | -4.788 | 1152.260 | 0.000 | -0.678 | -0.284 |
| fixed | F601 | -0.165 | 0.190 | -0.866 | 1431.355 | 0.387 | -0.538 | 0.209 |
| fixed | F631 | -0.122 | 0.152 | -0.800 | 1365.517 | 0.424 | -0.421 | 0.177 |
| fixed | F801 | 0.064 | 0.277 | 0.229 | 1266.856 | 0.819 | -0.480 | 0.607 |
| fixed | F811 | -0.102 | 0.131 | -0.780 | 1372.632 | 0.435 | -0.358 | 0.154 |
| fixed | F841 | -0.113 | 0.096 | -1.183 | 1326.594 | 0.237 | -0.302 | 0.075 |
| fixed | F901 | -0.005 | 0.099 | -0.052 | 1269.392 | 0.959 | -0.200 | 0.190 |
| fixed | F911 | -0.083 | 0.122 | -0.679 | 1414.897 | 0.497 | -0.322 | 0.156 |
| fixed | F921 | 0.172 | 0.115 | 1.488 | 1363.142 | 0.137 | -0.055 | 0.398 |
| fixed | F931 | 0.008 | 0.098 | 0.080 | 1432.654 | 0.936 | -0.185 | 0.201 |
| fixed | F941 | 0.059 | 0.146 | 0.402 | 1288.341 | 0.688 | -0.228 | 0.345 |
| fixed | F981 | -0.030 | 0.132 | -0.227 | 1427.611 | 0.820 | -0.289 | 0.229 |
| fixed | age | 0.093 | 0.016 | 5.938 | 1197.686 | 0.000 | 0.062 | 0.123 |
| fixed | bmi | 0.001 | 0.001 | 0.651 | 1140.015 | 0.515 | -0.001 | 0.003 |
| fixed | sexMALE | -0.146 | 0.080 | -1.835 | 1049.865 | 0.067 | -0.303 | 0.010 |

### MLR

| effect | term | estimate | std.error | statistic | df | p.value | conf.low | conf.high |
| --- | --- | --- | --- | --- | --- | --- | --- | --- |
| fixed | (Intercept) | 0.145 | 0.023 | 6.159 | 1219.151 | 0.000 | 0.099 | 0.191 |
| fixed | F121 | 0.038 | 0.022 | 1.703 | 1243.925 | 0.089 | -0.006 | 0.082 |
| fixed | F191 | 0.002 | 0.019 | 0.125 | 1429.933 | 0.900 | -0.034 | 0.039 |
| fixed | F231 | 0.037 | 0.021 | 1.798 | 1454.914 | 0.072 | -0.003 | 0.078 |
| fixed | F321 | 0.009 | 0.007 | 1.234 | 1415.459 | 0.217 | -0.005 | 0.023 |
| fixed | F331 | 0.006 | 0.020 | 0.285 | 1461.461 | 0.776 | -0.034 | 0.046 |
| fixed | F401 | -0.002 | 0.009 | -0.264 | 1421.534 | 0.792 | -0.021 | 0.016 |
| fixed | F411 | 0.007 | 0.013 | 0.537 | 1287.465 | 0.591 | -0.019 | 0.033 |
| fixed | F421 | 0.001 | 0.012 | 0.057 | 1328.628 | 0.954 | -0.022 | 0.024 |
| fixed | F431 | 0.001 | 0.011 | 0.138 | 1428.400 | 0.890 | -0.020 | 0.023 |
| fixed | F441 | 0.029 | 0.020 | 1.424 | 1348.318 | 0.155 | -0.011 | 0.069 |
| fixed | F451 | 0.003 | 0.015 | 0.198 | 1392.707 | 0.843 | -0.026 | 0.032 |
| fixed | F501 | -0.030 | 0.009 | -3.225 | 1200.387 | 0.001 | -0.049 | -0.012 |
| fixed | F601 | -0.007 | 0.018 | -0.399 | 1462.769 | 0.690 | -0.041 | 0.027 |
| fixed | F631 | 0.011 | 0.014 | 0.801 | 1401.969 | 0.423 | -0.016 | 0.039 |
| fixed | F801 | -0.014 | 0.026 | -0.530 | 1239.958 | 0.596 | -0.064 | 0.037 |
| fixed | F811 | -0.019 | 0.012 | -1.526 | 1381.668 | 0.127 | -0.042 | 0.005 |
| fixed | F841 | -0.003 | 0.009 | -0.369 | 1366.522 | 0.712 | -0.021 | 0.014 |
| fixed | F901 | 0.010 | 0.009 | 1.076 | 1296.555 | 0.282 | -0.008 | 0.028 |
| fixed | F911 | -0.004 | 0.011 | -0.367 | 1422.978 | 0.714 | -0.026 | 0.018 |
| fixed | F921 | 0.006 | 0.011 | 0.522 | 1395.798 | 0.601 | -0.015 | 0.027 |
| fixed | F931 | -0.006 | 0.009 | -0.695 | 1447.039 | 0.487 | -0.024 | 0.012 |
| fixed | F941 | 0.013 | 0.014 | 0.920 | 1317.615 | 0.358 | -0.014 | 0.039 |
| fixed | F981 | 0.008 | 0.012 | 0.654 | 1431.514 | 0.513 | -0.016 | 0.032 |
| fixed | age | 0.007 | 0.001 | 4.507 | 1207.228 | 0.000 | 0.004 | 0.009 |
| fixed | bmi | 0.000 | 0.000 | -0.028 | 1157.619 | 0.978 | 0.000 | 0.000 |
| fixed | sexMALE | 0.011 | 0.007 | 1.478 | 1051.078 | 0.140 | -0.004 | 0.026 |

### SIRI

| effect | term | estimate | std.error | statistic | df | p.value | conf.low | conf.high |
| --- | --- | --- | --- | --- | --- | --- | --- | --- |
| fixed | (Intercept) | 0.301 | 0.198 | 1.521 | 1157.165 | 0.129 | -0.087 | 0.689 |
| fixed | F121 | 0.602 | 0.189 | 3.179 | 1190.324 | 0.002 | 0.230 | 0.973 |
| fixed | F191 | -0.100 | 0.156 | -0.642 | 1424.274 | 0.521 | -0.407 | 0.206 |
| fixed | F231 | 0.458 | 0.175 | 2.619 | 1448.155 | 0.009 | 0.115 | 0.801 |
| fixed | F321 | 0.005 | 0.061 | 0.080 | 1405.368 | 0.937 | -0.115 | 0.125 |
| fixed | F331 | 0.072 | 0.172 | 0.418 | 1459.679 | 0.676 | -0.265 | 0.409 |
| fixed | F401 | -0.083 | 0.078 | -1.066 | 1413.779 | 0.286 | -0.236 | 0.070 |
| fixed | F411 | 0.037 | 0.110 | 0.339 | 1242.475 | 0.735 | -0.179 | 0.253 |
| fixed | F421 | -0.089 | 0.099 | -0.896 | 1298.026 | 0.371 | -0.284 | 0.106 |
| fixed | F431 | 0.051 | 0.090 | 0.565 | 1421.202 | 0.572 | -0.126 | 0.228 |
| fixed | F441 | 0.084 | 0.172 | 0.490 | 1320.692 | 0.624 | -0.254 | 0.423 |
| fixed | F451 | -0.046 | 0.126 | -0.366 | 1374.058 | 0.714 | -0.293 | 0.201 |
| fixed | F501 | -0.377 | 0.079 | -4.760 | 1141.102 | 0.000 | -0.533 | -0.222 |
| fixed | F601 | -0.232 | 0.148 | -1.569 | 1460.617 | 0.117 | -0.522 | 0.058 |
| fixed | F631 | -0.051 | 0.119 | -0.432 | 1389.007 | 0.666 | -0.285 | 0.182 |
| fixed | F801 | -0.053 | 0.218 | -0.242 | 1175.858 | 0.809 | -0.480 | 0.375 |
| fixed | F811 | -0.143 | 0.102 | -1.397 | 1358.689 | 0.162 | -0.343 | 0.058 |
| fixed | F841 | -0.111 | 0.075 | -1.474 | 1344.292 | 0.141 | -0.258 | 0.037 |
| fixed | F901 | 0.040 | 0.078 | 0.518 | 1254.432 | 0.605 | -0.113 | 0.193 |
| fixed | F911 | -0.041 | 0.095 | -0.429 | 1411.934 | 0.668 | -0.227 | 0.146 |
| fixed | F921 | 0.123 | 0.090 | 1.360 | 1380.548 | 0.174 | -0.054 | 0.300 |
| fixed | F931 | -0.020 | 0.077 | -0.268 | 1444.251 | 0.789 | -0.171 | 0.130 |
| fixed | F941 | 0.008 | 0.115 | 0.068 | 1280.242 | 0.946 | -0.217 | 0.233 |
| fixed | F981 | 0.025 | 0.103 | 0.239 | 1422.416 | 0.811 | -0.177 | 0.226 |
| fixed | age | 0.053 | 0.012 | 4.340 | 1142.566 | 0.000 | 0.029 | 0.077 |
| fixed | bmi | 0.001 | 0.001 | 1.530 | 1085.474 | 0.126 | 0.000 | 0.003 |
| fixed | sexMALE | -0.065 | 0.063 | -1.026 | 960.941 | 0.305 | -0.189 | 0.059 |
